# Changes in Urinary NGAL, FN, and LN Excretion in Type 2 Diabetic Patients Following Anti-Diabetic Therapy with Metformin

**DOI:** 10.3390/jcm14041088

**Published:** 2025-02-08

**Authors:** Anna Szeremeta, Agnieszka Jura-Półtorak, Alicja Grim, Kornelia Kuźnik-Trocha, Paweł Olczyk, Diana Ivanova, Yoana Kiselova-Kaneva, Krystyna Olczyk, Katarzyna Komosińska-Vassev

**Affiliations:** 1 Department of Clinical Chemistry and Laboratory Diagnostics, Faculty of Pharmaceutical Sciences in Sosnowiec, Medical University of Silesia, Katowice, Jedności 8, 41-200 Sosnowiec, Poland; ajura@sum.edu.pl (A.J.-P.); alicja.grim@gmail.com (A.G.); kkuznik@sum.edu.pl (K.K.-T.); olczyk@sum.edu.pl (K.O.); kvassev@sum.edu.pl (K.K.-V.); 2 Department of Community Pharmacy, Faculty of Pharmaceutical Sciences in Sosnowiec, Medical University of Silesia, Katowice, Jedności 10, 41-200 Sosnowiec, Poland; pawel.olczyk@sum.edu.pl; 3 Department of Biochemistry, Molecular Medicine and Nutrigenomics, Faculty of Pharmacy, Medical University “Prof. Dr. Paraskev Stoyanov”, 9002 Varna, Bulgaria; divanova@mu-varna.bg (D.I.); yoana.kiselova@mu-varna.bg (Y.K.-K.)

**Keywords:** NGAL, fibronectin, laminin, T2DM, obesity, metformin

## Abstract

**Background:** Excessive accumulation of glomerular extracellular matrix (ECM) is a key factor in the development and progression of diabetic nephropathy (DN). As kidney dysfunction has been reported in normoalbuminuric patients, identifying novel diagnostic and prognostic markers is essential for the prevention and treatment of DN. **Methods:** Urinary excretion of neutrophil gelatinase-associated lipocalin (NGAL) and ECM-related glycoproteins, i.e., fibronectin (FN) and laminin (LN), was measured in obese patients with newly diagnosed type 2 diabetes mellitus (T2DM) before and after 6 months of metformin therapy. **Results:** Baseline NGAL (1.27 (0.80–2.36) ng/mg Cr), FN (11.19 (5.31–21.56) ng/mg Cr) and LN (123.17 (54.56–419.28) pg/mg Cr) levels did not significantly differ between T2DM patients and controls (1.95 (1.09–2.97) ng/mg Cr, 11.94 (7.78–18.01) ng/mg Cr and 157.85 (83.75–326.40) pg/mg Cr, respectively). In multivariate regression analysis, the body mass index was identified as the only significant predictor influencing urinary NGAL and FN levels at baseline, with β = 0.249, *p* = 0.005 and β = 1.068, *p* = 0.010, respectively. Metformin treatment significantly increased urinary levels of both ECM proteins, i.e., FN (18.48 (11.64–32.46) ng/mg Cr) and LN (179.51 (106.22–414.68) pg/mg Cr), without any effect on NGAL levels (1.44 (0.81–2.72) ng/mg Cr). FN and LN were positively associated with NGAL both before (r = 0.709 and r = 0.646, both *p* < 0.001, respectively) and after (r = 0.594 and r = 0.479, both *p* < 0.001, respectively) therapy. No correlations were found between NGAL, FN, LN, and albuminuria. However, NGAL was positively correlated with the albumin/creatinine ratio (ACR) both before (r = 0.323, *p* < 0.05) and after (r = 0.287, *p* < 0.05) therapy, and negatively with estimated glomerular filtration rate (eGFR) in pre-treatment diabetics (r = −0.290, *p* < 0.05). FN and LN were also correlated with ACR (r = 0.384, *p* < 0.01 and r = 0.470, *p* < 0.001), although the association for LN was limited to untreated patients (r = 0.422, *p* < 0.01). **Conclusions:** Our results suggest that metformin has a beneficial effect on ECM turnover with a significant increase in urinary excretion of non-collagenous markers of glomerular injury, i.e., FN and LN. Additionally, ECM-related markers may serve as useful tools for monitoring early renal injury in obese diabetic patients.

## 1. Introduction

Type 2 diabetes mellitus (T2DM) is a chronic and progressive endocrinopathy associated with a higher risk of microvascular and macrovascular complications, which contribute to elevated diabetes-related morbidity and mortality rates. Both genetic and environmental factors contribute to the development of T2DM [1]. Nevertheless, the overweight, obesity, and a lack of physical activity are the strongest diabetogenic agents. Previous epidemiologic studies revealed that individuals with a higher body mass index (BMI) are more likely to develop type 2 diabetes compared to those with normal body weight [2,3]. Unhealthy obesity, characterized by dysfunctional adipose tissue, promotes a low-grade inflammatory state, leading to insulin resistance, the development of T2DM, and extracellular matrix (ECM) disorders [2]. Changes in ECM accumulation, composition, and organization lead to diabetes-related complications such as diabetic nephropathy (DN) [4]. Metformin, a synthetic biguanide, is one of the safest and most effective anti-hyperglycemic agents employed as first-line therapy for T2DM [5]. Previous clinical trials and experimental studies have shown that in addition to lowering blood sugar and body weight, metformin exerts renoprotective effects in diabetic kidney disease [5,6,7,8]. The mechanism by which metformin decreases the risk of renal impairment is not fully explained yet. Its effect is predominantly based on the induction of autophagy and suppression of inflammation, oxidative stress, and fibrosis [5,7,8,9]. However, the use of metformin is limited because of its potential lactic acidosis-related adverse effects, particularly in patients with severe renal impairment (defined as an eGFR < 30 mL/min/1.73 m^2^) [7,9].

Although urinary albumin excretion is generally considered the gold standard for early detection of renal impairment, it has limitations as changes in the glomerular basement membrane structure may occur before the appearance of microalbuminuria [10]. It is well known that 30% of patients with DN have normal urine albumin levels [11]. Furthermore, many non-renal factors, such as exercise, fever, infection, increase in blood pressure, or cardiac failure, are associated with a temporary increase in urinary albumin excretion [12]. Therefore, it is important to find alternative urinary biomarkers of diabetic renal injury, which would play a significant role in the optimal clinical care of type 2 diabetic patients.

Since plasma and urine levels of neutrophil gelatinase-associated lipocalin (NGAL) increase rapidly after tubular damage, NGAL is generally accepted as a useful early biomarker reflecting the severity of renal failure caused by diabetic disease [11,13,14]. NGAL, also known as lipocalin-2, siderocalin, or uterocalin, is a 25 kDa glycoprotein secreted mostly by activated neutrophils and also by kidney tubular cells, epithelial cells, and hepatocytes in response to inflammation and bacterial infection. Multiple biological roles have been ascribed to lipocalin-2, including iron-trafficking and chemotactic and bacteriostatic capacities [15,16,17]. It also has a protective effect on damaged renal tubules, stimulating the proliferation and epithelial differentiation of rat embryonic kidney cells. Given the relationship between NGAL and matrix metalloproteinase 9 (MMP-9), a protein involved in ECM remodeling, NGAL’s role in profibrotic mechanisms has also been suggested [15,16].

The most characteristic morphological change of diabetic kidney injury is the excessive synthesis and deposition of extracellular matrix proteins in glomerular basement membrane (GBM) and mesangium, leading to interstitial fibrosis and diffuse glomerulosclerosis. Diffuse GBM thickening is one of the earliest detectable feature of DN and may occur in almost all diabetic patients, irrespective of the presence of albuminuria. Increased expressions of specific predominate ECM glycoproteins, such as fibronectin (FN) and laminin (LN) have been documented following glomerular injury [4,18,19,20]. However, little is known about the relation between glomerular structural changes and the urinary excretion of FN and LN in diabetic patients.

Fibronectin is a high-molecular weight cell-adhesion glycoprotein widely distributed throughout the cell surface, plasma, and ECM. The plasma form of FN is synthesized primarily by hepatocytes and then secreted into the blood, whereas cellular FN is produced by various cell types, including fibroblasts, endothelial cells, and mesangial cells [21,22]. Fibronectin can bind to cell surface receptors called integrins and to the ECM via collagens, fibrin, and heparan sulfate proteoglycans (e.g., syndecans). The interactions between FN and integrins and syndecans promote several cellular processes, such as cell adhesion, growth, migration, and differentiation. FN is also involved in embryogenesis, wound healing, and blood clotting. Most importantly, fibronectin is essential for the normal organization and deposition of collagen by fibroblasts [22]. Thus, high glucose-induced FN overexpression may promote the aberrant mesangial accumulation of fibrillary collagen, potentially leading to renal fibrosis and, finally, to renal dysfunction or even renal failure.

Unlike fibronectin, the distribution of laminin, another non-collagenous glycoprotein of ECM, is primarily restricted to basement membranes, specifically the basal laminae [20,21]. Laminin shares several biological properties with FN. For example, LN, as a major adhesive protein of GBM [20], also interacts with the cell surface and other ECM components during organogenesis and differentiation [23]. Moreover, LN is involved in BM assembly and stability and plays a significant role in immune infiltration and inflammation. Interestingly, LN has been reported to stimulate the release of neutrophil extracellular traps by neutrophils, which must rapidly migrate from the circulation to sites of tissue injury or infection through the activated vascular BM [24].

However, despite numerous studies, the potential usefulness of urinary excretion of fibronectin and laminin as biomarkers of diabetic renal injury remains unclear. Thus, the main objective of the present study was to quantitatively evaluate NGAL as an early predictor of acute kidney injury and to assess the levels of two non-collagenous matrix glycoproteins—fibronectin and laminin—in the urine of obese patients with T2DM following anti-diabetic therapy with metformin.

## 2. Materials and Methods

### 2.1. Study Population

The study involved 53 normoalbuminuric (<30 mg/g) obese patients with early-onset type 2 diabetes defined as a disease duration ≤ 1 year. The cohort included 36 females and 17 males, aged from 44 to 72 years. T2DM was diagnosed according to the 2017 Polish Diabetes Association criteria: fasting plasma glucose (FPG) ≥ 126 mg/dL (≥7.0 mmol/L); or glycaemia at 120 min during an oral glucose tolerance test (OGTT) ≥ 200 mg/dL (≥11.1 mmol/L). The following exclusion criteria were applied: (1) age < 18 years or >75 years; (2) type 1 diabetes or other specific types of diabetes; (3) pregnancy or lactation; (4) malignancies; (5) autoimmune diseases; (6) serious comorbidities such as heart, kidney, liver disorders; (7) hyperthyroidism and other endocrine diseases; (8) treatment with glycocorticosteroids, adrenocorticotropic hormone, or interferons. All subjects received 500–850 mg of metformin three times a day with meals for 6 months. At both baseline and after 6 months follow-up, patients underwent standard anthropometric measurements, including height, weight, waist circumference (WC), waist-to-hip ratio (WHR), and blood pressure (BP). Fasting blood samples were drawn for determination of glucose, insulin, glycated haemoglobin (HbA1C), total cholesterol (TC), high- and low-density lipoprotein cholesterol (HDL-C and LDL-C), triglycerides (TG), creatinine, and cystatin C (cysC). Furthermore, albumin and creatinine levels were measured in the first-morning urine samples.

The study also included twenty-six age- and sex-matched overweight/obese individuals without diabetes as a control group. The controls were recruited based on the following eligibility requirements: absence of any history of medical or surgical diseases; absence of hospital admissions during the previous 3 years; no smoking and no using any type of medication; and results of morphological and biochemical analyses within the reference range. All obese participants exhibited the metabolically healthy (MHO) phenotype, characterized by normal insulin sensitivity, low prevalence of hypertension, and favorable glucose and lipid metabolism parameters. The anthropometric and laboratory measurements of the obese healthy subjects and T2DM patients are presented in Table 1.

The study was approved by the Bioethical Committee of the Medical University of Silesia in Katowice (KNW/0022/KB1/147/10). All subjects provided informed consent prior to their inclusion in the study, and the research was carried out in accordance with the relevant guidelines and regulations of the Declaration of Helsinki.

### 2.2. Biological Material

After 8–10 h of fasting, venous blood and spot urine samples were collected from each participant. In diabetic patients, samples were obtained twice: at baseline and 6 months after starting metformin treatment, while for the control group—samples were collected only once. Venous blood was collected into heparin-treated tubes and centrifuged for 10 min at 1500× *g* at room temperature. After routine diagnostic tests, the remaining plasma was immediately stored in small aliquots at –80 °C. Urine samples were collected in sterile disposable containers, centrifuged at 1500× *g* for 15 min at 4 °C to remove urinary sediment, including whole cells and debris. The resulting urine supernatant was transferred to 2.0 mL Eppendorf tubes and frozen at −80 °C until further analysis.

### 2.3. Anthropometric Determination

Each participant’s height was measured to the nearest 0.5 cm with a wall-mounted stadiometer, and body weight was measured to the nearest of 0.5 kg using a digital glass scale (Beurer, GS49, Ulm, Germany). BMI was calculated as weight (kg) divided by square of height (m^2^). WC was assessed midway between the lower costal margin and the iliac crest with an inflexible measurement tape. Hip circumference was measured at the widest point around the buttocks. Systolic and diastolic blood pressures were taken with a standardized sphygmomanometer after at least 5 min of rest.

### 2.4. Laboratory Blood and Urine Analyses

Routine biochemical parameters including markers of glycemic variability, lipid profile, and serum creatinine, were measured using an automated blood analyzer, following methods described in our earlier investigations [25]. Measures of renal function were determined by serum creatinine and cystatin C and estimated glomerular filtration rate (eGFR) was calculated by the Modification of Diet in Renal Disease (MDRD) equation. Urine albumin to creatinine ratio (UACR) was used to classify patients as having normoalbuminuria (UACR < 30 µg/mg), microalbuminuria (UACR 30–299 µg/mg), and macroalbuminuria (UACR ≥ 300 µg/mg). UACR was determined from the first-morning urine sample. Serum cystatin C levels were assayed by a commercial sandwich-type enzyme-linked immunosorbent assay (ELISA) kit from BioVendor R&D (Brno, Czech Republic) according to the producer’s recommendations. The analytical detection limit for cystatin C was 0.25 ng/mL. All patient samples measurements were conducted in a single run and intra-assay coefficients of variation were 3.4%.

### 2.5. Pancreatic Islet Cell Function

In all participants, the homeostasis model assessment 2 (HOMA2) was used to estimate insulin resistance (HOMA2-IR), insulin secretory function (HOMA2-%S), and β-cell function (HOMA2-%β) indices. These were calculated with a HOMA calculator released by the Diabetes Trials Unit, University of Oxford: HOMA Calculator (available at http://www.dtu.ox.ac.uk./homacalculator). The quantitative insulin-sensitivity check index (QUICKI) was also calculated using the following equation QUICKI = 1/[log (fasting insulin, µU/mL) + log (fasting glucose, mg/dL)]. Insulin resistance was also calculated using the estimated glucose disposal rate (eGDR) according to the following formula: eGDR (mg/kg/min) = 24.31 − 12.22 (WHR) − 3.29 (hypertension 0/1) − 0.57 (HbA1c). eGDR value < 7.5 (mg/kg/min) was considered to be an indicator of reduced tissue sensitivity to insulin.

### 2.6. Immunoassay of NGAL and Non-Collagenous Matrix Glycoproteins—Fibronectin and Laminin

The assessment of NGAL and fibronectin concentrations in urine samples was performed using a commercially available sandwich ELISA kit from BioVendor R&D (Brno, Czech Republic) according to the manufacturer’s instructions. However, laminin concentration was determined using an ELISA kit from Cloud-Clone Corporation (Katy, TX, USA). The minimum detectable concentrations were estimated to be =0.02 ng/mL for NGAL, =0.1 ng/mL for FN, and <3.2 pg/mL for LN. All patient’s samples were assayed in duplicate on the same day, and intra-assay coefficients of variation were <7.9% for NGAL, <6% for FN, and <10% for LN.

### 2.7. Statistical Analysis

The statistical analyses were performed using the Statistica 13.3 software (TIBCO Software Inc., Palo Alto, CA, USA). The normality of distribution of continuous variables was tested by the Shapiro–Wilk test. Continuous variables with normal distribution were presented as mean ± standard deviation (SD); non-normal variables were reported as median and interquartile (25th–75th percentile) range. The assumption of the homogeneity of variances was tested using Levene’s test. Comparisons between parameters in patients with T2DM and controls were performed using Student’s *t*-test or the Mann–Whitney U test. Student’s *t*-test or the Wilcoxon signed rank test were used to assess changes in parameters within paired samples of each T2DM patient. The percentage changes in urinary NGAL, FN, and LN from before to after metformin therapy were calculated. Spearman’s test was used for correlation analysis. Multiple regression analysis of the data was also performed. A *p*-value was considered significant if it was <0.05. In addition, the details for the statistical analyses of each result are presented in the corresponding table or figure legends.

## 3. Results

### 3.1. Renal Function

There were no significant differences in urinary albumin, urinary, and serum creatinine levels or urinary albumin-to-creatinine ratio (ACR) and eGFR between healthy obese individuals and T2DM patients (Table 2). Furthermore, the level of these parameters remained within the normal range after 6 months of metformin therapy. However, a significantly higher serum cystatin C level was found in obese, newly diagnosed diabetic patients before the implementation of metformin treatment, as compared to control subjects (Table 2). Interestingly, continued metformin therapy led to the normalization of cystatin C levels in T2DM patients (Table 2).

### 3.2. Urinary Excretion of NGAL, FN, and LN

**Figure 1:**
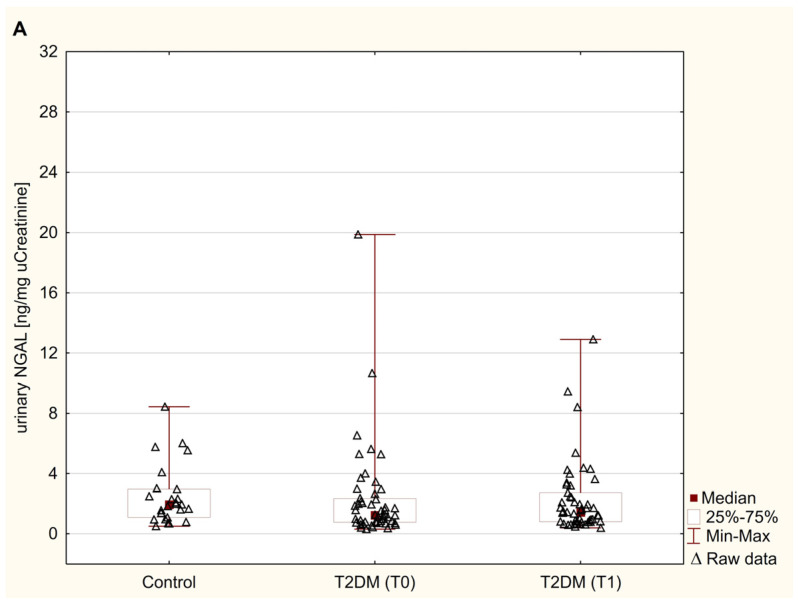

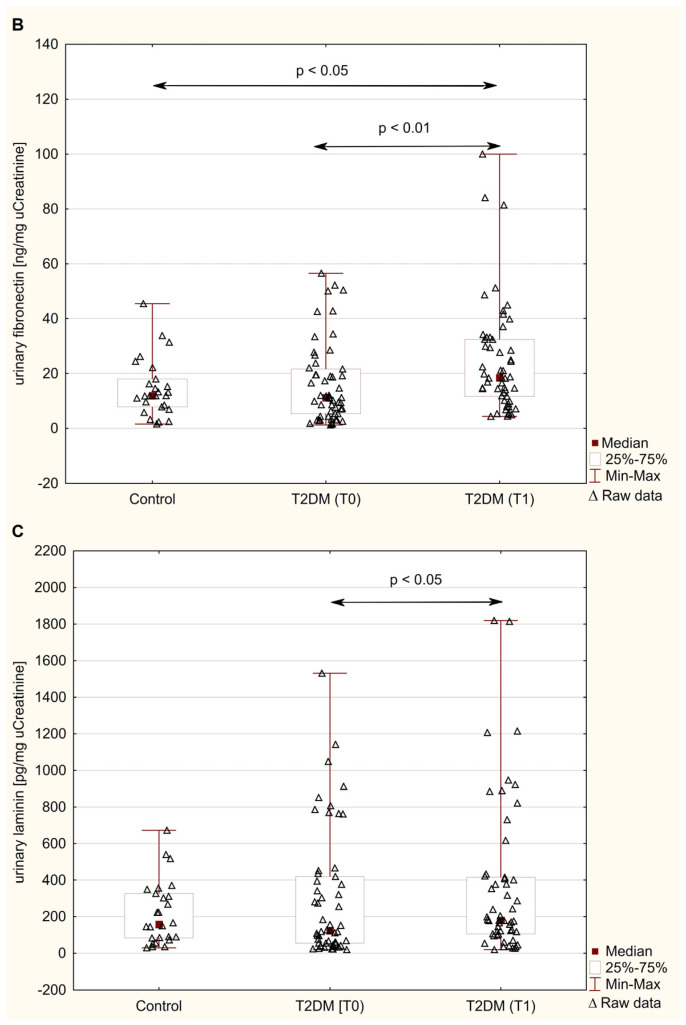
Urinary levels of NGAL (**A**), FN (**B**), and LN (**C**) in control subjects and T2DM patients before and after 6 months of metformin therapy. Results are expressed as median, inter-quartile (25th–75th percentile) range. Statistical significance was determined using the Mann–Whitney U test or the Wilcoxon signed rank test. A *p*-value was considered significant if it was <0.05. FN, fibronectin; NGAL, neutrophil gelatinase-associated lipocalin; LN, laminin; T_0_, baseline; T_1_, 6 months after metformin therapy; T2DM, type 2 diabetes mellitus.

The distribution of urinary NGAL, FN, and LN excretions in obese patients with type 2 diabetes undergoing metformin therapy and non-diabetic control are presented in Figure 1A–C. The data showed that the urinary excretion of NGAL and extracellular matrix proteins, i.e., FN and LN in diabetic patients before metformin therapy did not differ from the controls (*p* = 0.116, *p* = 0.855, and *p* = 0.921, respectively; Figure 1A–C). Moreover, urinary NGAL in T2DM patients did not show any differences during 6 months of metformin therapy (*p* = 0.673; Figure 1A). On the other hand, treatment with metformin led to a significant increase in the urinary levels of FN [18.48 (11.64–32.46) ng/mg creatinine] and LN (179.51 (106.22–414.68) pg/mg creatinine), compared to baseline values (11.19 (5.31–21.56) ng/mg creatinine, and 123.17 (54.56–419.28) pg/mg creatinine, respectively), (*p* < 0.01 and *p* < 0.05, respectively; Figure 1B,C). Furthermore, at 6 months, urinary excretion of FN in T2DM patients increased by 55% compared to the results obtained in healthy subjects (*p* < 0.05; Figure 1B), whereas LN excretion was still not significantly different from the control values (*p* = 0.245; Figure 1C).

### 3.3. Correlation Analysis of NGAL, FN, and LN with Kidney Function Parameters

The analysis of the relationship between urinary NGAL and matrix proteins (FN and LN), as well as selected parameters of kidney function (urinary albumin, ACR, eGFR, and serum creatinine) in untreated diabetic patients and after 6 months of metformin therapy, is shown in Table 3 and Table 4.

A significant positive correlation between the concentration of NGAL and urinary FN and LN before (r = 0.709 and r = 0.646, both *p* < 0.001, respectively; Table 3) as well as after (r = 0.594 and r = 0.479, both *p* < 0.001, respectively; Table 3) 6 months of treatment with metformin in T2DM patients was detected. No correlations between NGAL, FN, and LN levels and the urinary concentration of albumin were noted (Table 4). However, the data showed that NGAL was positively correlated with ACR both before (r = 0.323, *p* < 0.05, Table 4) and after (r = 0.287, *p* < 0.05, Table 4) initiation of metformin therapy, and negatively with eGFR (r = −0.290, *p* < 0.05, Table 4) in pre-treatment obese diabetic patients. Similarly, FN showed a positive correlation with ACR both before (r = 0.384, *p* < 0.01, Table 4) and after (r = 0.470, *p* < 0.001, Table 3) 6 months of metformin therapy. In the case of LN, the analysis showed a significant positive correlation with ACR (r = 0.422, *p* < 0.01) only among untreated diabetic patients, whereas eGFR showed no association with LN in the same group (r = −0.003 *p* = 0.984, Table 4). No significant association between the evaluated urinary parameters and serum creatinine and cystatin C was found. Furthermore, there was a positive correlation between percentage change in urinary NGAL excretion and percentage change in the concentration of FN and LN (r = 0.619 and r = 0.566, both *p* < 0.001, respectively; Table 5).

A multivariate linear regression analysis was performed to assess the relationship between urinary excretion of NGAL, FN, and LN in T2DM patients at baseline, considering relevant variables such as BMI and systolic arterial blood pressures that may have influenced the parameters assessed. This analysis showed that BMI was the only variable in the model that had significant impact on the levels of urinary excretion of NGAL and FN in patients at baseline (β = 0.249, *p* = 0.005, and R2 model = 0.162; β = 1.068, *p* = 0.010, and R^2^ model = 0.153, respectively; Table 6).

## 4. Discussion

### 4.1. Urinary Excretion of NGAL in Obese T2DM Patients Before Metformin Therapy

In this study, urinary excretion of NGAL was analyzed in obese patients with type 2 diabetes undergoing metformin therapy and non-diabetic control. The results indicate that urinary NGAL levels in diabetics were unaffected by treatment and did not differ significantly from those in obese healthy individuals, both before and after 6 months of metformin therapy. These findings contradict prior studies [26,27,28,29,30,31,32,33] suggesting NGAL as a reliable marker of early renal impairment. Previous research has reported that NGAL increases in both serum and urine before the presence onset of microalbuminuria in diabetic patients [27,29,30,32]. However, our study focused on newly diagnosed T2DM cases, none of whom presented with microalbuminuria. Urinary NGAL was found to be affected by disease duration, severity, and the presence of comorbidities that could lead to DN [14,26,27,34]. Most previously published studies [26,27,28,29,30,31,32] evaluating urinary lipocalin-2 excretion were conducted among patients with diabetes duration longer than six years. For example, in the study of Al-Hazmi et al. [26], urinary NGAL values were observed across healthy controls and patients with T2DM whose mean disease duration ranged from 10 to 15 years. Urinary NGAL levels were strongly associated with reduced renal function, and increased in all diabetic groups compared to non-diabetic control [26]. Kaul et al. [28] reported similar results in T2DM patients, with uNGAL significantly increased from normoalbuminuric to macroalbuminuric patients, and was positively correlating with urinary albumin excretion. Furthermore, uNGAL levels were higher in normoalbuminuric patients than in healthy subjects, indicating that early tubular injury may develop as a result of metabolic and hemodynamic stress associated with chronic hyperglycemia [28]. On the other hand, some research did not show the same level of sensitivity for uNGAL, as it was not significantly increased in normoalbuminuric diabetic patients [35,36,37]. Similar to our study, Kim et al. [35] did not find any difference between the control and normoalbuminuric groups. Additionally, in a study conducted by Yiğit Kaya et al. [38] among 38 T2DM patients with a disease duration of not less than 5 years, they found similar uNGAL levels in diabetic patients and healthy subjects, stating that urinary NGAL does not have a potential diagnostic value for the early detection of DN. Importantly, in this study [38], age, BMI, glycemic control, and eGFR were similar to our study. Finally, in accordance with our results, Greco et al. [39] did not observe significant changes in either plasma and urinary NGAL levels between newly diagnosed normoalbuminuric T2DM patients without DN and metabolically healthy, non-obese controls. As mentioned earlier, these inconsistencies in uNGAL levels across studies may reflect differences in disease severity, age at onset, diabetes duration, presence of inflammation, and glycemic control status. NGAL is known to regulate the inflammation, acting as an acute phase protein and chemokine [15]. Moreover, it has been shown that both adipocyte mRNA expression of NGAL and its circulating levels increase in obesity, suggesting a link between NGAL and insulin resistance and glycolipid metabolism [15,16,17,40]. In line with this, the upregulated expression of NGAL has been reported in several murine obese/diabetic models as well as human [13,15]. Interestingly, also a study by Rashad et al. [41] reported increased levels of serum and urine NGAL in non-diabetic obese patients compared with non-obese controls. Moreover, the circulating NGAL were positively associated with adiposity, hyperglycemia, and hypertriglyceridemia in patients with metabolic and heart diseases [40]. Despite its potential relevance, the precise role of NGAL in obesity pathogenesis remains unclear. NGAL influences the peroxisome proliferator-activated receptors γ (PPARγ) activity, which regulates lipogenesis, adipocytokine synthesis, and insulin signaling [17]. In our study, the lack of significant differences in urinary NGAL levels between newly diagnosed T2DM patients and healthy subjects likely reflects high BMI in both groups. Interestingly, T2DM patients in our study belonged to the obesity class category, while subjects in the control group belonged to the unique subgroup of obese individuals with normal metabolic status, known as metabolically healthy obese. Approximately 10–25% of obese people are metabolically healthy due to preserved insulin sensitivity and are more resistant to obesity-associated metabolic disorders [42]. However, the impact of MHO phenotype on renal function remains debated. While studies [43] suggest that obesity increases the risk of kidney dysfunction in MHO subjects, others report no such association [44].

Numerous studies that have used BMI to evaluate obesity on the progression of renal function in T2DM have demonstrated that higher BMI can promote the occurrence and progression of diabetic kidney disease (DKD) [45,46]. However, other studies have suggested that elevated BMI is a protective factor against renal impairment and may be associated with an increased survival rate of patients with end-stage renal disease (ESRD) [46,47]. These discrepancies may be explained to the confounding factors. Besides hyperglycemia and obesity, hypertension, dyslipidemia, and cardiovascular disease play important roles in DKD progression as well [46]. Furthermore, while the distribution, rather than the overall amount of adipose tissue critically influences DKD onset, BMI may not be an accurate measure of obesity in T2DM patients. Most notably, BMI is affected not only by fatness but also other body components, such as musco-skeletal mass and body fluid [47,48,49]. Therefore, the kidney burden of obesity may be underestimated by relying on BMI. While BMI differed significantly between the groups of our study, it is worth noting that the waist–hip ratio (WHR)—a more distinct measure of central adiposity and visceral fat—was comparable between the control and diabetic groups. Given that central obesity is closely linked to metabolic complications and renal outcomes, this consistency in WHR supports the validity of the comparative analyses. However, no significant association between uNGAL and WHR was found.

Given the asymptomatic onset of obesity-associated renal disease, further studies are needed to elucidate the role of uNGAL in the early detection of renal changes in obese, newly diagnosed T2DM patients. Nevertheless, the present study confirm that urinary NGAL levels positively correlated with ACR, both before and after metformin therapy initiation, and negatively with eGFR in pre-treatment diabetic patients. These relationships align with observations in diabetic [26] and non-diabetic [41] obese. Fu et al. [33] also observed significant correlations between uNGAL, ACR, and eGFR in T2DM patients, including normoalbuminuric cases, suggesting NGAL as a potential marker for early renal impairment in short-term T2DM [33]. However, conflicting results regarding uNGAL’s diagnostic utility have been published [36,38].

Due to the potential relationship between NGAL levels and obesity-related metabolic complications, we also evaluated cystatin C, another biomarker of kidney function, in obese patients with type 2 diabetes and subjects with MHO. Serum cysC levels are primarily dependent on eGFR and are not significantly affected by non-renal factors such as muscle mass, weight, and nutritional status [50]. Cystatin C-based GFR estimates have been shown to predict all-cause mortality, cardiovascular events, as well as kidney failure in diabetic and obese patients [51,52]. In this study, normoalbuminuric obese diabetic patients exhibited significantly higher serum cysC levels than obese controls, suggesting a certain degree of renal dysfunction not detected by creatinine, NGAL, or eGFR measurements. This elevation correlated with increasing ACR and decreasing eGFR, consistent with previous findings [13]. These results highlight the superiority of cysC over creatinine and uNGAL in detecting early kidney dysfunction.

### 4.2. Urinary Excretion of Non-Collagenous Matrix Glycoproteins (FN and LN) in Obese T2DM Patients Before Metformin Therapy

Fibronectin, known for its multiple adhesive properties and its role in the recruitment of fibroblasts and accumulation of other extracellular matrix components [22], is one of the first ECM proteins present during early lesions of mesangial expansion [21]. Immunohistochemical studies have demonstrated increased FN deposition in diabetic kidney biopsies correlating with glomerular abnormalities [53,54,55,56]. However, it remains unclear whether the increased FN in injured glomeruli reflects local synthesis or transfer from the bloodstream [55]. Interestingly, studies have shown elevated production of FN by human mesangial cells in exposed to high glucose concentrations, suggesting that hyperglycemia may directly induce FN synthesis or inhibit its degradation [57]. Conflicting data exist regarding soluble FN levels in microalbuminuric patients with diabetes, with some studies reporting higher plasma levels of FN in diabetic patients [58,59,60], while other studies did not [61,62]. Higher urinary FN levels have been found in diabetic patients especially those with macroalbuminuria [63,64]. Further evidence supporting the predictive role of FN in early renal injury includes higher urinary excretion of FN in microalbuminuric patients compared to normoalbuminuric controls [65,66].

These aforementioned findings suggest that urinary FN excretion increases as a consequence of progressive kidney tissue damage caused by diabetes. Despite growing evidence linking fibronectin with the development of glomerulosclerosis in DN, the present study did not find significant differences in urinary FN levels between newly diagnosed obese T2DM patients and controls. However, a strong positive correlation was observed between urinary FN and uNGAL both before and after 6 months of metformin therapy. Additionally, a moderate positive association was found between FN and urinary albumin excretion. Similar observations have been described by Muh Anshar and colleagues [67]. Their study found no change in urinary FN excretion between microalbuminuric T2DM patients and non-diabetic subjects, as well as no significant relationship between FN and urine albumin levels [67]. Muh Anshar et al. [67] also did not find any statistically significant difference in FN levels between T2DM patients with diabetic nephropathy (micoalbuminuric group) and those without DN (normoalbuminuric group). Moreover, Orih et al. [65] presented evidence indicating a relationship between urinary FN levels and the degree of albuminuria. They found a significant positive correlation between FN and microalbuminuria, as well as between FN and ACR [65], indicating the potential clinical application of urinary FN as a complementary marker for early kidney damage.

In relation to the second non-collagenous matrix glicoprotein analyzed in this study, i.e., laminin, we noted findings similar to those of urinary FN. The urinary LN levels were not significantly different in the obese patients with newly diagnosed type 2 diabetes compared with control subjects. However, we found positive correlations between urinary LN and ACR, as well as tubular dysfunction markers, such as uNGAL in the diabetic group. To the best of our knowledge, no previous studies have analyzed the laminin concentrations in the urine of obese patients with newly diagnosed diabetes, and thus we cannot directly compare our findings with those of other authors. Nevertheless, our results differ from those of Banu et al. [68], Hayashi et al. [69], and Miyake et al. [70], who demonstrated that urinary LN excretion is higher in diabetic subjects compared to healthy controls, even prior to the onset of microalbuminuria, suggesting that LN could be a marker for predicting albuminuria. Furthermore, Banu et al. [68] revealed significantly higher laminin to albumin excretion ratios in T2DM patients with diabetic nephropathy compared to non-diabetic nephropathy patients, and found a positive relationship between urinary LN and tubular dysfunction markers, such as N-acetyl-β-D-glucosaminidase and α-1 microglobulin. These findings, in line with the strong positive correlation between urinary LN and NGAL levels in patients with newly diagnosed type 2 diabetes found in this study, indicate that urinary excretion of laminin in diabetics is linked with both glomerular and tubular dysfunctions and/or damage. Similar results were described by Orih et al. [65], who found higher urinary levels of LN in patients with microalbuminuria compared to healthy controls, indicating diabetic glomerular damage in the early stage of kidney dysfunction. In agreement with our findings, Orich et al. [65] also reported a positive correlation between urinary LN and ACR in T2DM patients. A positive correlation was also observed between urinary LN and FN levels and microalbuminuria. Even more importantly, ROC analysis showed that urinary LN had good diagnostic values for early prediction of diabetic kidney disease and could substitute albuminuria in the routine evaluation of T2DM patients [65].

The absence of increased urinary excretion of non-collagenous markers of glomerular injury, i.e., fibronectin and laminin in T2DM patients found in the present study, is not easily explained. This discrepancy may stem from differences in patient group categorization. In our study, newly diagnosed obese diabetic patients were normoalbuminuric with normal eGFR and without signs of insulin resistance or dyslipidemia. In contrast, diabetic patients in previous studies were typically not obese, exhibited varying levels of albuminuria, and had longer disease duration. What is equally important, most of the studies concerning the assessment of urinary excretion of FN and LN in diabetic patients relate the obtained results to the excretion of these proteins in the urine of non-obese healthy individuals. Therefore, further studies are needed to support the role of fibronectin and laminin as prognostic markers of early kidney injury in obese T2DM patients.

### 4.3. The Influence of 6 Months of Metformin Therapy on the Urinary Excretion of NGAL and Non-Collagenous Matrix Glycoproteins (FN and LN)

In this study, after 6 months of metformin therapy, conventional biomarkers of kidney function such as urinary albumin, urinary and serum Cr, as well as ACR and eGFR remained within the normal range in obese T2DM patients. In addition, serum cystatin C levels significantly decreased, reaching values similar to those in metabolically healthy obese individuals, indicating metformin’s protective effects on renal function. However, in contrast to cysC, the urinary levels of novel biomarkers of renal injury such as NGAL, remained unchanged following metformin therapy. While no comparable data are available for T2DM patients, studies on other populations, such as diabetic patients undergoing carotid endarterectomy, Eilenberg et al. [71] have shown that metformin reduces inflammatory state through the reduction of NGAL serum levels, and thus lowers the risk of cerebral embolic events.

Regarding fibronectin and laminin, the study revealed that metformin therapy led to a significant increase in their urinary levels after 6 months. These results may reflect changes in kidney function, as both glycoproteins showed positive correlations with ACR and uNGAL. This could support the hypothesis that metformin alters the deposition and turnover of ECM proteins. Excessive extracellular matrix accumulation in diabetic renal tissues does not directly correlate with clinical injury [10]. Although early GMB thickening may occur without clinical signs like increased albuminuria or reduced eGFR [10], it is associated with altered ECM synthesis and/or reduced degradation [4,18,72]. Metformin’s effect on ECM turnover may be linked to its ability to modulate hyperglycemia-induced metabolic pathways. Chronic exposure of cultured human or murine mesangial cells to a high glucose environment can induce the generation of reactive oxygen species, trigger the polyol pathway, induce the activity of protein kinase C, enhance expression of transforming growth factor β (TGF-β), and promote the formation of early and advanced glycation end products (AGEs) [4,18,73]. These last ones are formed by irreversible cross-linking of glucose with ECM structural proteins, including type IV collagen, fibronectin, laminin, and proteoglycans [74]. Most importantly, AGEs disrupt the balance between synthesis and degradation of glomerular basement membrane ECM components, especially through reduced activity of MMPs [74], which are key in ECM degradation [20,75]. Therefore, the increased urinary excretion of fibronectin and laminin in T2DM patients during antidiabetic therapy seem to result from a metformin’s beneficial effect on ECM turnover and renal fibrosis. Indeed, metformin appears to attenuate ECM over-deposition and renal fibrosis in a model of adenine-induced renal injury in mice [76], prevent epithelial–mesenchymal transition (EMT) in high glucose-treated renal tubular epithelial cells in vitro [77], and reduce cyclosporine A-induced renal fibrosis in rats [78]. Furthermore, a study conducted by Wang et al. [77] revealed that metformin can reduce renal tubulointerstitial fibrosis, especially in the early stage of DN, with a significant increase in autophagy and a decrease in the expression of fibrotic biomarkers, such as fibronectin and type I collagen in kidney tissue. Autophagy is an evolutionarily conserved lysosomal protein degradation pathway that plays a key role in the removal of damaged cytoplasmic organelles and abnormal proteins to maintain cellular homeostasis under stress conditions. Dysregulation or absence of autophagy has been observed in diabetic kidney disease, and restoration of this process is a new therapeutic strategy against the progression to diabetes-related complications [79,80]. In general, metformin treatment induces autophagy via several signaling pathways, including AMP-activated protein kinase (AMPK) signaling and AMPK-independent pathways [8,81]. AMPK is a well-known cellular energy sensor responsible for switching from anabolic to catabolic metabolism to regulate energy homeostasis [8]. By stimulating AMPK, metformin helps prevent inflammatory responses, modulate oxidative stress by inhibiting reactive oxygen species production, regulate cell differentiation, and influence pathological processes in various diseases, including T2DM, providing therapeutic benefits [82,83]. Additionally, animal model studies have demonstrated metformin attenuates renal interstitial fibrosis by suppressing the TGF-β1/Smad2/3 pathway, which plays an essential role in gene expression, including encoding ECM proteins [9,76]. Metformin has also been shown to suppress TGF-β1-induced inflammatory responses and inhibit the expression of type IV collagen and fibronectin in human proximal tubular cells [84]. In summary, these findings confirm that metformin plays a critical role in renal protection and tubulointerstitial fibrosis by attenuating over-deposition of ECM proteins.

### 4.4. Limitations and Strengths of the Study

There were some limitations to this study. First, it is only an observational cohort study rather than a randomized controlled trial. Second, the study group was relatively small (but homogenous) and included a well-defined cohort of obese patients with normoalbuminuria and early T2DM (diagnosed within <1 year). This population was carefully selected based on strict inclusion and exclusion criteria, ensuring homogeneity and reducing variability associated with comorbidities or other confounding conditions. Third, the lack of a control group without obesity or subjects with a similar BMI but without diabetes is a limitation. While obesity alone can impact renal biomarkers, such as NGAL and ECM-related glycoproteins, we selected MHO individuals as controls to minimize variability from metabolic dysfunction and focus on the effects of early T2DM. However, future studies with broader control groups, including non-obese individuals and metabolically unhealthy obese patients, are needed to better understand the complex interplay between diabetes, obesity, and renal biomarkers. However, the obtained results provide a foundational basis for further research, where a larger, randomized cohort will be incorporated to validate and expand upon our initial findings.

## 5. Conclusions

To our knowledge, this study is the first to report increased levels of ECM-related markers, specifically the most abundant non-collagenous glycoproteins of GBM—fibronectin and laminin—in the urine of obese, newly diagnosed patients with T2DM during anti-diabetic therapy with metformin. These changes may indicate that the renoprotective effect of metformin is mediated by the attenuation of excessive extracellular matrix protein deposition. Moreover, the positive correlations between urinary levels of FN and LN and markers of renal function, such as ACR and uNGAL, indicate a potential clinical application of urinary ECM-related markers to monitor early renal injury in obese diabetic patients. In addition, the absence of elevated urinary NGAL levels in obese T2DM patients compared to metabolically healthy obese controls suggests that uNGAL is not a useful marker for assessing renal function in obese, newly diagnosed T2DM patients with normoalbuminuria.

## Figures and Tables

**Table 1:** The characteristic of control subjects and T2DM patients before and after 6 months of metformin therapy.

Parameter	Control (*n* = 26)	Patients with T2DM (*n* = 53)			
		Baseline (T_0_)	6 Months After Metformin Therapy (T_1_)	*p*-Values C vs. T_0_	*p*-Values T_0_ vs. T_1_
Gender female/male (n)	12 F/14 M	36 F/17 M			
Age (years)	56.00 (52.00–60.00)	59.00 (53.00–65.00)		0.106	
Weight (kg)	82.50 (76.00–89.00)	85.00 (80.00–96.00)	84.00 (79.00–97.00)	0.085	**0.010**
Height (cm)	170.13 ± 8.85	164.50 ± 9.45		**0.015**	
BMI (kg/m^2^)	28.40 (26.11–30.83)	31.43 (30.13–34.41)	31.24 (29.05–34.52)	**<0.001**	**0.010**
Waist (cm)	99.00 (88.00–104.50)	105.00 (101.00–109.00)	103.00 (99.00–110.00)	**<0.001**	**0.003**
Hip (cm)	102.00 (98.00–106.00)	108.00 (103.00–116.00)	107.00 (101.00–115.00)	**<0.001**	**<0.001**
WHR (cm^2^)	0.98 (0.89–1.01)	0.97 (0.92–1.03)	0.98 (0.92–1.020)	0.431	0.641
SABP (mmHg)	120.00 (115.00–120.00)	137.50 (130.00–145.00)	130.00 (125.00–137.50)	**<0.001**	**<0.001**
DABP (mmHg)	80.00 (70.00–85.00	80.00 (80.00–85.00)	80.00 (70.00–82.50)	0.347	**0.021**
Glucose (mg/dL)	90.00 (86.00–96.00)	117.50 (110.00–124.50)	115.00 (106.00–123.00)	**<0.001**	0.356
HbA_1c_ (%)	5.15 ± 0.36	6.42 ± 0.73	6.37 ± 0.81	**<0.001**	0.534
Insulin (µIU/mL)	7.30 (4.00–17.70)	7.65 (3.10–12.85)	7.65 (4.00–10.25)	0.770	0.191
HOMA-IR2	1.33 (0.52–2.11)	1.10 (0.67–1.85)	1.20 (0.73–1.70)	0.739	0.670
HOMA-S (%)	113.10 (47.80–255.00)	90.45 (54.45–183.50)	85.10 (68.60–162.60)	0.389	0.771
HOMA-B (%)	85.80 (57.50–160.45)	60.40 (35.80–087.90)	59.70 (36.45–78.95)	**0.009**	0.774
Index QUICKI (%)	0.35 (0.32–0.41)	0.34 (0.32–0.39)	0.34 (0.32–0.37)	0.396	0.760
eGDR (mg/kg/min)	9.74 (8.94–10.28)	8.12 (6.64–9.23)	8.70 (7.15–9.74)	**<0.001**	**0.004**
Cholesterol (mg/dL)	193.00 (173.00–223.00)	188.00 (163.00–222.00)	184.00 (170.00–206.00)	0.714	0.180
HDL-C (mg/dL)	46.00 (41.00–52.00)	48.70 (40.40–55.70)	50.00 (41.20–59.40)	0.527	0.077
LDL-C (mg/dL)	116.00 (103.00–148.00)	117.90 (85.60–141.40)	101.60 (77.40–124.00)	0.999	**0.008**
no-HDL-C (mg/dL)	145.00 (122.00–174.60)	142.00 (121.60–174.90)	140.50 (115.80–157.00)	0.860	0.259
Triglicerydes (mg/dL)	136.00 (107.00–176.00)	120.00 (99.00–184.00)	119.00 (94.00–174.90)	0.680	0.434
TG/HDL-C	3.38 (1.95–4.03)	2.45 (1.94–4.04)	2.48 (1.76–3.74)	0.615	0.205

Results are expressed as mean ± SD or median, inter-quartile (25th–75th percentile) range, or percentage (%). Comparisons between parameters in control subjects and patients at baseline were performed using Student’s *t*-test or the Mann–Whitney U test. Student’s *t*-test or the Wilcoxon signed rank test were used to assess changes in parameters within paired samples of each T2DM patient. A *p*-value was considered significant if it was <0.05. Bold value is significant at *p* < 0.05. BMI, body mass index; C, control; DAPB, diastolic arterial blood pressures; eGFR, estimated glomerular filtration rate; eGDR, glucose disposal rate; F, female; HbA_1c_, glycosylated haemoglobin A_1c_; HDL-C, high-density lipoprotein cholesterol; HOMA-B, homeostatic model assessment of B cell function; HOMA-IR2, homeostatic model assessment of insulin resistance; HOMA-S, homeostatic model assessment of insulin sensitivity; Index QUICKI, quantitative insulin sensitivity check index; LDL-C, low-density lipoprotein cholesterol; M, male; no-HDL-C, no high-density lipoprotein cholesterol; SD, standard deviation; SABP, systolic arterial blood pressures; T_0_, baseline; T_1_, 6 months after metformin therapy; T2DM, type 2 diabetes mellitus; TG, triglicerydes; WHR, waist–hip ratio.

**Table 2:** Parameters for assessing kidney function of control subjects and T2DM patients before and after 6 months of metformin therapy.

Parameter	Control (n = 26)	Patients with T2DM (n = 53)		*p*-Values	
		Baseline (T_0_)	6 Months After Metformin Therapy (T_1_)	C vs. T_0_	T_0_ vs. T_1_
sCr (mg/dL)	0.86 (0.77–0.91)	0.82 (0.71–0.89)	0.83 (0.73–0.92)	0.070	**0.006**
eGFR (mL/min.1.73m^3^)	91.00 (82.00–100.00)	95.00 (85.40–108.20)	90.00 (76.00–105.00)	0.188	**0.004**
Cystatin C (µg /mL)	1.11 (0.98–1.28)	1.33 (1.13–1.47)	1.15 (1.00–1.31)	**0.002**	**<0.001**
uAlbumin (µg/mL)	2.50 (2.50–6.40)	3.90 (2.50–9.70)	2.80 (2.50–6.90)	0.257	0.656
uCr (mg/dL)	102.50 (64.00–129.00)	101.00 (73.00–169.00)	92.00 (70.00–151.00)	0.549	0.598
ACR (µg/mg Cr)	5.00 (2.89–6.43)	5.43 (3.05–8.04)	4.31 (2.92–7.30)	0.375	0.556

Results are expressed as median, inter-quartile (25th–75th percentile) range. Statistical significance was determined using the Mann–Whitney U test or the Wilcoxon signed rank test. A *p*-value was considered significant if it was <0.05. Bold value is significant at *p* < 0.05. ACR, creatinine and albumin/creatinine ratio; C, control; sCr, serum creatinine; eGFR, estimated glomerular filtration rate; s, serum; T_0_, baseline; T_1_, 6 months after metformin therapy; T2DM, type 2 diabetes mellitus; u, urinary.

**Table 3:** Correlations between urinary levels of NGAL, FN, and LN in T2DM patients before and after 6 months of metformin therapy.

Parameter	Patients with T2DM (n = 53)			
	Baseline (T_0_)		6 Months After Metformin Therapy (T_1_)	
	NGAL (ng/mg Cr)			
	r	*p*	r	*p*
FN (ng/mg Cr)	0.709	**<0.001**	0.594	**<0.001**
LN (ng/mg Cr)	0.646	**<0.001**	0.479	**<0.001**

Spearman’s test was used for correlation analysis. Bold value is significant at *p* < 0.05. Cr, creatinine; FN, fibronectin; NGAL, neutrophil gelatinase-associated lipocalin; LN, laminin; r, Spearmen correlation coefficient; T_0_, baseline; T_1_, 6 months after metformin therapy; T2DM, type 2 diabetes mellitus.

**Table 4:** Correlations between urinary levels of NGAL, FN, and LN, and the other kidney function parameters of T2DM patients before and after 6 months of metformin therapy.

Parameter	Patients with T2DM (n = 53)			
	Baseline (T_0_)		6 Months After Metformin Therapy (T_1_)	
	NGAL (ng/mg Cr)			
	r	*p*	r	*p*
sCr (mg/dL)	0.056	0.699	−0.063	0.659
Cystatin C (µg /mL)	0.120	0.498	0.022	0.904
eGFR (mL/min.1.73m^3^)	−0.290	**0.039**	−0.182	0.200
uAlbumin (µg/mL)	−0.049	0.740	−0189	0.188
ACR (µg/mg Cr)	0.323	**0.023**	0.287	**0.046**
	FN (ng/mg Cr)			
sCr (mg/dL)	−0.091	0.519	−0.086	0.540
Cystatin C (µg /mL)	0.118	0.493	0.112	0.517
eGFR (mL/min.1.73m^3^)	−0.053	0.706	−0.069	0.624
uAlbumin (µg/mL)	0.032	0.826	−0.074	0.600
ACR (µg/mg Cr)	0.384	**0.005**	0.470	**<0.001**
	LN (ng/mg Cr)			
sCr (mg/dL)	0.010	0.945	0.225	0.105
Cystatin C (µg /mL)	0.081	0.640	0.372	**0.026**
eGFR (mL/min.1.73m^3^)	−0.003	0.984	−0.188	0.178
uAlbumin (µg/mL)	0.102	0.475	−0.050	0.723
ACR (µg/mg Cr)	0.422	**0.002**	0.208	0.143

Spearman’s test was used for correlation analysis. Bold value is significant at *p* < 0.05. ACR, albumin/creatinine ratio; sCr, serum creatinine; eGFR, estimated glomerular filtration rate; FN, fibronectin; NGAL, neutrophil gelatinase-associated lipocalin; LN, laminin; r, Spearmen correlation coefficient; T_0_, baseline; T_1_, 6 months after metformin therapy; T2DM, type 2 diabetes mellitus; u, urinary.

**Table 5:** Correlations between the percentage change in urinary NGAL and the percentage changes in urinary FN and LN, and the other kidney function parameters of T2DM patients.

Parameter	Patients with T2DM (n = 53)	
	% Change NGAL	
	r	*p*
% change FN	0.619	**<0.001**
% change LN	0.566	**<0.001**
% change sCr	0.040	0.783
% change Cystatin C	−0.082	0.646
% change eGFR	0.017	0.905
% change uAlbumin	−0.040	0.608
% change ACR	0.273	0.061

The calculation of percentage changes was made as follows: (value post-value pre-intervention)/value pre-intervention × 100. Spearman’s test was used for correlation analysis. Bold value is significant at *p* < 0.05. ACR, albumin/creatinine ratio; sCr, serum creatinine; eGFR, estimated glomerular filtration rate; FN, fibronectin; NGAL, neutrophil gelatinase-associated lipocalin; LN, laminin; r, Spearmen correlation coefficient; T2DM, type 2 diabetes mellitus; u, urinary.

**Table 6:** Multiple linear regression analysis for predictors of baseline urinary NGAL, FN, and LN levels in T2DM patients.

Parameter	Patients with T2DM (n = 53) at Baseline (T_0_)		
	NGAL (ng/mg Cr)		
	β	*p*	Total Predict Model
BMI (kg/m^2^)	0.249	**0.005**	R^2^: 0.162; estimated R^2^: 2.968
SABP (mmHg)	0.025	0.481	*p* = **0.016**
	FN (ng/mg Cr)		
BMI (kg/m^2^)	1.068	**0.010**	R^2^: 0.153; estimated R^2^: 13.824
SABP (mmHg)	−0.212	0.200	*p* = **0.017**
	LN (ng/mg Cr)		
BMI (kg/m^2^)	12.802	0.203	R^2^: 0.070; estimated R^2^: 346.219
SABP (mmHg)	−5.732	0.166	*p* = 0.178

Bold value is significant at *p* < 0.05. FN, fibronectin; NGAL, neutrophil gelatinase-associated lipocalin; LN, laminin; SABP, systolic arterial blood pressures; T2DM, type 2 diabetes mellitus.

## Data Availability

Data are contained within the article.
